# Beyond accreditation: unraveling the narrative of public health through a critical race praxis lens

**DOI:** 10.3389/fpubh.2024.1383077

**Published:** 2024-11-08

**Authors:** Sarah L. Collins, Acquel Allen-Mitchell, Travis C. Smith, George Hack, Nichole E. Stetten, Michael D. Moorhouse

**Affiliations:** ^1^Department of Health, Sport & Exercise Sciences, School of Education and Human Sciences, University of Kansas, Lawrence, KS, United States; ^2^College of Public Health and Health Professions, University of Florida, Gainesville, FL, United States; ^3^Department of Educational Foundations, Leadership, and Technology, College of Education, Auburn University, Auburn, AL, United States; ^4^Department of Occupational Therapy, College of Public Health and Health Professions, University of Florida, Gainesville, FL, United States; ^5^School of Public Health, Survey Research Center, Brown University, Providence, RI, United States

**Keywords:** Critical Race Theory, public health, curriculum, equity, qualitative methods, Public Health Critical Race Praxis

## Abstract

**Introduction:**

Recognizing and addressing health inequities among minority populations are pivotal to public health. Further, public health strives to understand the complexities between race and health without limiting discussions around race as a trivial variable. This commitment toward equity demonstrates considerable similarities to Critical Race Theory (CRT) which led to the creation of the Public Health Critical Race (PHCR) Praxis to instill CRT within public health. However, the literature on how public health education incorporates critical race studies remains limited. The goal of this study was to examine how public health curriculum currently aligns with the PHCR praxis and meets public health's goal of health equity.

**Methods:**

This qualitative study employed document analysis to evaluate academic syllabi from CEPH-accredited MPH programs. Stratified random sampling was applied across two sampling pools, Schools of Public Health (SPH), and Public Health Programs (PHP). Course overviews, course objectives, course curricular information, and course policies were identified and extracted from each syllabus for analysis. A total of 53 syllabi were obtained from a final sample of nine public universities and one private.

**Results:**

Through inductive and directed content analysis, a priori themes of Structural Determinism, Voice, Critical Approaches, Ordinariness of Racism, Social Construction of Knowledge, Intersectionality, Disciplinary Self-Critique, Primacy of Racialization, Race as a Social Construct, Race Consciousness, and their respective categories arose as salient. Two new themes, Antiracism Practices and Culture of Inclusivity, were also present.

**Discussion:**

This study is the first to explore public health education's current curricular practices concerning CRT and antiracist praxes. The results confirm the interwoven nature of public health education with critical race studies, as all principles of PHCR praxis were present. However, the prevalence of these principles varied, suggesting gaps in the alignment of public health curricula and CRT. It is essential that public health educators ensure that the foundational competencies students are expected to display align with public health's goal of health equity. This work can equip MPH programs and public health educators with the ability to revise or bolster their current curricular and instructional efforts to support the pursuit of health, racial equity, and social justice.

## 1 Introduction

Identifying, attending to, and reducing ethnic and racial minority health disparities and inequities has served as a cornerstone of public health for the past 40 years, yet little evidence exists demonstrating how critical race studies have been integrated within public health education and training. Public health, arguably more than any other health profession, prepares students to respond to social change and understand the unique and complex health challenges encountered by racial and ethnic minorities (1). As such, public health programs and schools of public health must train students to not only think of race and ethnicity as nominal variables, but rather dedicate an overt and explicit criticality to the conversation.

However, the way schools and programs of public health can meet this goal remains elusive. The Council of Education for Public Health (CEPH), the accrediting body for schools and programs of public health, introduced “diversity” as a new accreditation criterion in 2011 (2, 3). This criterion mandates schools and programs of public health demonstrate a commitment toward diversity through various programmatic levels and practices, including curriculum. As Artinian et al. (4) state, to provide high-quality care for a continually diversifying population, it is essential that health care and public health professionals be educated in environments that value diversity.

One of the most prominent and leading underpinnings within diversity discussions across scholarly discourse is Critical Race Theory (CRT). Originally conceived out of Critical Legal Studies, CRT embodies a philosophical approach, conceptual and analytical framework, and intellectual and social movement (5–8). CRT's central premise focuses on dismantling and eliminating racism rather than race (9), thus making it relevant to a vast array of micro interactions and macro systems. To attend to this mission, scholars and activists have collectively committed to studying and transforming the current relationship between race, racism and power to ultimately achieve an antiracist society (10). To fulfill this commitment, CRT articulates several tenets for race scholars and activists to operate within, including: racism is ordinary, not aberrational, race is socially constructed, interest convergence, differential racialization, intersectionality, and voice-of-color or counter-stories (10). Complementary to these tenets, CRT invites a corresponding construct to the conversation—antiracism. CRT and antiracism are inextricably interwoven with the clear objective to emancipate individuals and communities from oppressive systems and environments based on the premise of race. Antiracism could be characterized as the manifestation of CRT ideologies into active practices that dismantle racist policies, systems, behaviors, beliefs, etc. (11, 12).

Similar to how antiracism and CRT are operationally intertwined, public health and CRT share an innate compatibility due to public health's overarching commitment toward reducing and eliminating health disparities and inequities among minoritized and marginalized populations. Though they are indistinguishably interwoven, public health and CRT have not been widely discussed or published in tandem. However, in recognition of the dearth of race criticality within public health, Ford and Airhihenbuwa (13, 14) developed the Public Health Critical Race (PHCR) Praxis—the first public health framework to speak directly to and infuse CRT within public health operations. The authors note that this work paralleled Ladson-Billings and Tate's original efforts within K-12 (15, 16), utilizing CRT to address racism's contribution to educational disparities, in that there was no applicable framework that utilized CRT within a public health context to prioritize equity of health outcomes (17). This cutting-edge framework is composed of four overarching foci and ten respective principles. The four foci represent key domains within CRT and public health work, noting an interdependent, overlapping and reciprocal relationship between *Contemporary Patterns of Racial Relations, Knowledge Production, Conceptualization & Measurement*, and *Action*. These foci are accompanied by ten affiliated principles, which include: primacy of racialization, race as a social construct, ordinariness of racism, structural determinism, social construction of knowledge, critical approaches, intersectionality, disciplinary self-critique, voice, and race consciousness (13).

The dissemination of the PHCR praxis has catalyzed a notable shift within research practices (18) however public health education continues to be limited within these conversations. A previous study found only 18 examples from peer-reviewed literature of curricular, pedagogical, or instructional practices and strategies that integrated critical theories of race (19). Among those, utilized theories and methodologies varied ranging from a presentation of the unique perspective of being both doctoral students and college instructors, who sought to link the content being discussed in their core public health courses to legacies of racism, colonialism, and other structural determinants of health as a means to evolve with student needs, societal momentum, and program commitments (20) to a systematic review that identified public health programs, curricula, and pedagogical methods that reify structural racism as a contributing factor to health disparities, social inequities, and structural issues (21). Though all warranted and needed to continue advancing public health education and CRT discussions forward, individuals continue to provide comment that more work is needed (22).

As such, due to the minimal work overtly dedicated toward investigating the role of CRT within public health education, this study aims to be the first to investigate public health education's curricular practices in relation to CRT and anti-racist praxes. More specifically, this study will conduct a document analysis to evaluate CEPH-accredited MPH programs' ability to satisfy CEPH's diversity criterion within public health curriculum (2, 3, 23). The following research questions will be addressed: (1) How are instructors implicitly integrating and explicitly presenting racial tenets and antiracist principles in their academic syllabi within Master of Public Health courses? (2) Which racial tenets and antiracist principles are the most prevalent within public health course syllabi? (3) Where are the racial tenets and antiracist principles positioned within the course syllabus? Establishing public health's curricular baseline in meeting CEPH's diversity competency is not only essential for developing informed action items to alter and adapt current curricular, pedagogical, and instructional practice, but also serves as a disciplinary self-critique to illustrate potential discrepancies and inconsistencies in public health educational approaches toward advancing health equity. Thus, this study is vital to not only assess our achievement CEPH's competency, but also toward accomplishing public health's mission of social justice and antiracism within our programs.

## 2 Methods

This qualitative study utilized document analysis as a methodological means to conduct evaluation research. Document analysis has been cited on several occasions as a methodological means to conduct evaluation research (24, 25) and is described as a systematic methodologic procedure for identifying, collecting and analyzing documents for their relevance and significance (26, 27). Academic syllabi represent a communication document and are often a student's first means of engaging with a course and therefore, should be considered a primary data source that has the potential to elicit rich information about a program's curriculum. Thus, this study evaluated academic syllabi from CEPH-accredited Master of Public Health (MPH) programs using procedures described below.

### 2.1 Data collection

CEPH-accredited MPH programs were identified through (1) Schools of Public Health (SPH) and (2) Public Health Programs (PHP), as categorized on the CEPH website (28). The researchers intentionally maintained this separation to allow for comparison between accreditation qualifications, with the interest in uncovering whether that those who have an entire school dedicated to CEPH accreditation may be more mindful toward holistically addressing components of the PHCRP compared to individual programs. However, the reverse may also be true that those with only one program may inherently be more detail-oriented in their approach, prioritizing the PHCRP principles throughout their program initiatives compared to an entire SPH. Thus, two separate, alphabetized lists were developed respective to their accreditation category (i.e., SPH or PHP) to serve as the sampling pools. Using systematic random sampling procedures, a random number generator was used to establish the starting point within each sample frame, followed by selecting a random number between 1–10 to establish an a priori sampling interval (29). Since there are currently no standards for qualitative sample size, the researchers utilized current literature to identify an optimal number of units to achieve saturation across the data (30, 31). The researchers assessed this number by comparing SPH and PHP to focus group discussions, due to the parallel nature of syllabi representing individuals and the collection of them within an MPH imitating a “focus group.” Thus, based upon the interval criteria, the researcher identified five units from each sampling pool.

Each unit's public website was reviewed for an MPH curriculum overview to identify each program's “core” didactically-grounded courses. Once core classes were identified, a program representative was contacted via email introducing the researcher, outlining the overarching aim of this study, and requesting the most up-to-date identified core MPH academic syllabi. Program representatives were also notified that all academic syllabi would be deidentified, including removal of course code, course name, and instructor information to maintain confidentiality and anonymity of institution, prior to data analysis. Within 1 week of unresponsiveness, a follow-up reminder email was sent. Upon another week of unresponsiveness, the unit was removed from the sampling frame and the researcher re-sampled from the original lists, excluding the previously selected SPH/PHP. This process occurred until five SPH and five PHP were identified, and their respective MPH core course syllabi were collected. In instances when some core course syllabi were accessible, a SPH/PHP was only retained if at least half of their reported core course syllabi were obtained. The recruitment process was conducted from March through May 2022. In accordance with the CEPH accreditation procedures (32), all identified units were required to meet the original 2011 diversity criterion. However, six of the ten unit were also reaccredited under the updated 2016 standards, which included an additional competency, “Discuss the means by which structural bias, social inequities, and racism undermine health and create challenges to achieving health equity at organizational, community, and societal levels” (33) through annual reporting requirements and/or reaccreditation standards.

Following the completion of data collection, the researchers engaged in data cleaning to establish a higher degree of trustworthiness (34), and ensure consistency across multiple coders. The following four sections of the course syllabi were identified for analysis: course overview, course objectives, course curricular information (i.e., instructional strategies, course content, etc.), and course policies, as these sections have been recommended and cited as common syllabi components (35, 36). Data were organized according to the four analysis sections, labeling the various pieces of data by their unique code.

### 2.2 Data analysis

**Figure 1:**
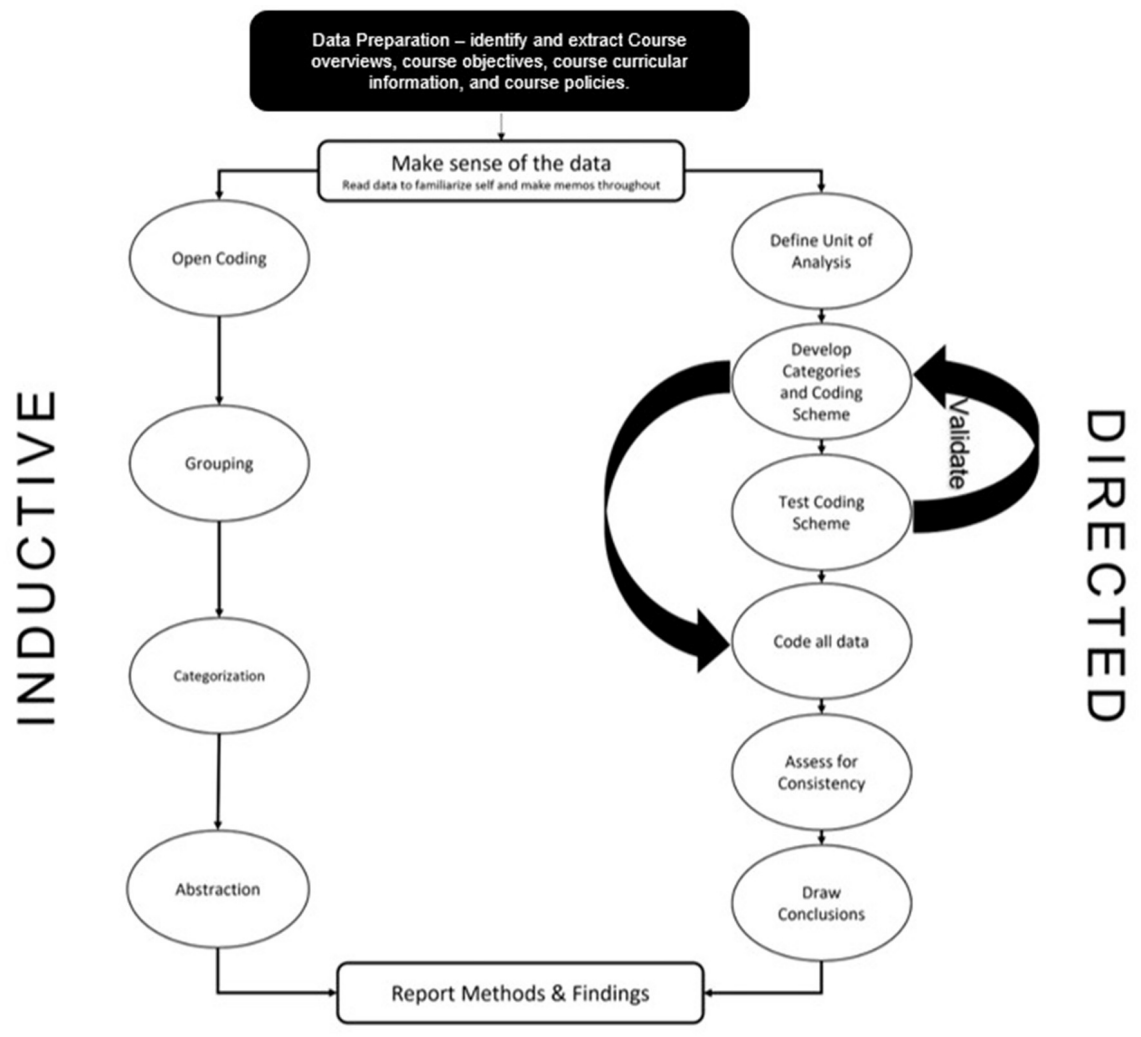
Data analysis procedures.

Inductive and directed content analysis were used due to their ability to investigate objectively and systematically (37, 38), and evaluate the data quantitatively through frequency of words and/or phrases, allowing meaning and significance to be assessed through prevalence (39). Directed content analysis utilizes deductive coding processes to analyze data within a theoretical framework, ultimately seeking to validate or expand upon the existing framework (40). Zhang and Wildemuth's (41) directed coding procedures were used. The categories and coding scheme were developed using the Public Health Critical Race (PHCR) praxis. The PHCR praxis is ultimately defined as a methodological tool for public health research, therefore minor adaptations were made to be more relevant to public health education. For salient themes that did not coincide with the developed coding scheme, inductive coding procedures were used. A visual representation of all analysis procedures is presented in Figure 1. All data analysis was conducted in NVivo 12 Plus software application.

First, two coders independently analyzed the data using the described directed and inductive coding procedures. At the time of analysis, the two coders were doctoral candidates in a PhD Public Health program, with research agendas centered around racial health equity. One coder was an alumnus of an MPH program, while the other was actively pursuing their MPH in conjunction with their PhD. Both coders identified as female, with one identifying as Black and the other as White. One of the researchers specialized in their training to include exploration and investigation into CRT, as well as its complimentary racial frameworks such as TribalCrit, AsianCrit, LatCrit, and Intersectionality. The researcher describes this training as a transformative experience, vowing to center and prioritize all of their public health efforts to include a CRT mindset and lens.

In accordance with Zhang and Wildemuth's (41) procedures, the coders met to negotiate their findings after analyzing only the SPH data. During the negotiations, the coders either validated the a priori operational definitions used in the coding scheme or made minor revisions to better reflect the data. After these intermediate negotiations, the coders resumed analysis procedures for the PHP data. Once the coders finished their independent review, they met for a final round of negotiations. Again, negotiations continued until 100 percent consensus was met.

## 3 Results

The participation of five SPH and five PHP was acquired throughout the 2-month recruitment period. Resampling procedures were required due to several selected institutions either being unresponsive (*n* = 8), declining to participate (*n* = 1), or not having an MPH (*n* = 1). The final sample of institutions was composed of nine public universities and one private. The locations of the institutions included the Midwest (*n* = 4), Northeast (*n* = 2), Southeast (*n* = 2), and Southwest (*n* = 2). Thirty one and 22 syllabi were obtained from the participating PHPs and SPHs, respectively.

All a priori established themes were present within the data. In addition to the established codebook, two additional themes and two new categories were identified as salient throughout. Results are presented by theme in descending order of frequency. Corresponding categories and differences between prevalence of themes within PHPs and SPHs are discussed within their respective sections. A summary of frequencies of each theme and category, as well as their distribution across syllabus sections are provided in Tables 1, 2.

**Table 1:** Theme and category frequencies.

**Theme**	**Category**	**CEPH-accreditation identification**		**Total**
		**Public health programs**	**Schools of public health**	
Structural determinism		166	76	242
Antiracism practice		123	31	154
Voice		61	41	102
	Co-Construction of Knowledge	83	59	142
Ordinariness of racism		52	25	77
Critical approaches		51	36	87
	Advocacy	23	34	57
Intersectionality		49	18	67
Culture of inclusivity		48	34	82
Social construction of knowledge		37	34	71
Disciplinary self-critique		28	22	50
Primacy of racialization		24	7	31
Race as a social construct		18	5	23
Race consciousness		2	1	3

**Table 2:** Theme and category distribution across syllabus sections.

**Theme**	**Category**	**Syllabus section**			
		**Course competencies, objectives & goals**	**Course overviews**	**Curricular information**	**Policies**
Structural determinism		107	21	112	2
Antiracism practice		29	7	70	48
Voice		32	10	52	8
	Co-construction of knowledge	8	4	94	36
Ordinariness of racism		28	6	33	10
Critical approaches		26	6	54	1
	Advocacy	19	7	28	3
Intersectionality		7	1	27	32
Culture of inclusivity		0	3	5	74
Social construction of knowledge		48	4	19	0
Disciplinary self-critique		29	5	16	0
Primacy of racialization		11	1	17	2
Race as a social construct		9	1	8	5
Race consciousness		0	1	2	0

### 3.1 Structural determinism

Structural determinism was an a priori theme defined as “the fundamental role of macro-level forces in driving and sustaining inequities across time and contexts; the tendency of dominant group members and institutions to make decisions or take actions that preserve existing power hierarchies” (13). No adaptations to the original definition were required because the PHCR praxis approach details utilizing a multilevel lens to include policy, social, and cultural aspects in addition to individual and interpersonal factors, an idea relevant to both research and education. Of note, recent literature has moved that there is an interrelated, yet distinct difference between *structural determinism* and *social determinants of health*. Both concepts were captured within this operationalization of “Structural Determinism,” thus encompassing a wide breadth of multilevel factors, but with varying depictions of analysis of *power* within these systems.

This theme was the most frequently identified theme in both PHPs and SPHs with 166 and 76 accounts, respectively. Often it was found that a single syllabus or institution would emphasize the prioritization of structural determinism by repeating or rephrasing a similar sentiment. For example, the use of CEPH's learning objective, “Discuss the means by which structural bias, social inequities and racism undermine health and create challenges to achieving health equity at organizational, community and societal levels,” was identified and noted to align with structural determinism's operational definition on several syllabi. Other examples included module or session titles such as, “Liberty, paternalism, coercion, and other principles,” “Social determinants of behavior and social change,” and “Policy decisions and powerful interests.” Others specifically included them as assignment prompts for students to directly engage with, “Students will identify a health outcome, associated risk factors at all levels of the [Social Ecological Model] SEM, and the morbidity and mortality of a specific population experiencing disparities that result from structural bias, social inequities, and racism.” One assignment prompt tethers how governing bodies and structural discrimination influence various dimensions of community wellness:

How did intergovernmental relations help produce the contamination of the drinking water in Flint, Michigan and the inadequate response to such a seemingly obvious public health threat? What is the capacity of Flint and other communities across the U.S. to protect and promote their physical and social wellbeing in the face of systemic racism?

Structural determinism was least prevalent within the policies across both the PHPs and SPHs, however, there was a distinct difference in where this theme was most emphasized between PHPs and SPHs. PHPs had an overwhelming majority of the structural determinism accounts within the course competencies, objectives, and goals, whereas SPHs had more within the curricular information.

### 3.2 Antiracism practices

Antiracism Practices was the second most commonly identified theme across the academic syllabi, with just under 80 percent of these accounts residing within the PHPs. Antiracism Practices was created from inductive coding procedures to encompass the salient and reoccurring nature of explicit statements, commitments, or demonstrations of antiracism. More specifically, the act of not only acknowledging racism's pervasiveness, but also an intentional effort to abolish it. This is distinct from the original PHCRP's “Action” focus in that it does not merely identify opportunities for action (13), but rather is an action itself. Within course objectives, this often manifested within objectives that specifically note an action-based approach such as “demonstrate,” “apply,” or “integrate.” These were all in relation to practicing race criticality associated with the other themes. In addition, some syllabi had overt language dedicated within the course overviews to preface the course activities. For example, one course overview stated,

In the MPH curriculum, several core courses teach how racism fuels the inequities that lead to health disparities. The MPH Program will work with partners to fortify the curriculum and look for learning opportunities outside the classroom to help students, staff, faculty, and the community to come together to tackle this vitally important issue.

Several courses directly point to specific frameworks in which the course will operate within such as, “[this] course will utilize a social justice perspective to examine both historical and current issues with a focus on the underlying causes of these injustices and solutions to those problems” and,

This course is designed to provide students with grounding in the social determinants of health through a health equity framework. Students will learn about promoting health equity within [state] communities and how to integrate health equity frameworks into their public health practice and research.

Within curricular information, one syllabus offered a more implicit, yet impactful practice of antiracism by having no class on holiday's not typically recognized within university calendars such as Yom Kippur and Succoth. Also within this section, several assigned readings directly spoke to antiracism such as, “Building a Social Justice Narrative for Public Health” (42) and “Public Health as Social Justice” (43), which draw a direct parallel between public health and social justice. Others within this section have their students directly engage with antiracism through assignment prompts: “If we can recognize those scripts, we have the opportunity to interrupt them, and potentially transform them before we negatively impact someone, or reify and reproduce inequality.”

Finally, course policies had several accounts of antiracism that depict clear expectations regarding course conduct. For example, “Exclusionary, offensive, or harmful speech (such as racism, sexism, homophobia, transphobia, etc.) will not be tolerated and in some cases may be subject to University harassment procedures.” Other examples of policies focused less on enforcement and more on relational accountability regarding antiracism:

Utilizing the institutional civility code we will promote the values of diversity, equity, and inclusion, both inside and outside our classrooms. To this end, SPH upholds the expectations that all course participants will acknowledge diverse experiences in the classroom, create environments that encourage equitable classroom participation, and ensure that students and faculty abide by SPH policies and procedures.

Among the exemplary models within this theme, there was several accounts of overlap with the inductively developed theme, Culture of Inclusivity, since the two concepts share a strong moral and epistemological grounding. Ultimately, both of these examples emphasize an overt dedication toward upholding antiracist ideals and practices within the classroom.

### 3.3 Voice

Voice encompasses prioritizing the perspectives and privileging the experiential knowledge of marginalized persons through presentations of alternative methodologies, counter narratives, explicit mention of using scholars of color, etc. It also entails the use of research and teaching methodologies as a potential mechanism for centering marginalized voices, presenting alternative epistemologies from traditional Eurocentric norms, and challenging traditional ontologies. This theme, though originally outlined within the PHCR praxis, also includes a new category titled, Co-Construction of Knowledge, to depict both the implicit and explicit recognition that student diversification offers. More specifically, student diversity offers unique perspectives and capitalizing on individual personal and professional experiences as a facet of constructing course discussion and knowledge. There were 102 accounts of syllabus language that fit within the general parameters of Voice and an additional 142 accounts specifically noted as Co-Construction of Knowledge.

Starting with course competencies, objectives and goals, a clear precedent was set to value and promote all voices. One program specifically outlined a learning objective to, “evaluate stakeholder interests, voices, values/goals, especially with regard to diverse and underserved populations,” while another outlined a key learning objective of, “discuss how empowerment is about building power with those most impacted,” both of which draw overt attention to this theme. Though Voice was present in all sections of the syllabi, it was most prevalent within the curricular information, such as weekly topics, assignment descriptions and readings, across both PHPs and SPHs. For example, one PHP had a week dedicated to Black women's health issues and specifically notes “raising community voices” as part of the topic title. This was then supported through a weekly assigned reading titled, “Commentary: Listening to Black Women: The Critical Step…” Within this example, the following week continued its mission toward health equity and centralizing Voice by having a week dedicated to “fostering conversations that help people in communities understand one another better,” in which “The Local Voices Network (LVN)” was used as an instructional tool. The LVN was described as “a unique physical-digital network designed to bring under-heard community voices, perspectives, and stories to the center of a healthier public dialogue” and was launched in 2019 by the PHPs local community. Utilizing this conversational network strategy not only promotes and prioritizes marginalized persons' experiential knowledge, but also offers an alternative method of instruction, potentially making the learning environment more accessible and equitable to a variety of learning styles. Other innovative instructional strategies were identified such as a syllabus asking students to,

Explore leadership by reading a leadership related book following your own interests. Perhaps you would like to read a book written by a current or recent past leader, such as: … The above list is by no means comprehensive of the types of books you may choose from - any book that you might like to explore to gain more insight into your personal leadership journey is appropriate and does not have to be a textbook. It can be a biography, or a historical account of a major leadership challenge.

This assignment depicts an intentionality to offer nontraditional forms of readings that potentially center alternative epistemologies and voices allowing students to explore a wider breadth of public health knowledge and experience. Some assignments were more global in their attention to Voice by requiring students to consider what “stakeholder*s*” were relevant to conversations, with particular attention to “community engagement” and “community members” as relevant stakeholders. This, in turn, prioritized the perspectives and experiential knowledge of those we serve rather than traditional ontologies and epistemologies. Across the various syllabus sections, policies had the lowest count of Voice present. However, one exemplary model of attending to Voice within policies was a SPH's commitment to supporting diversity, equity and inclusion by promoting “Brave (rather than safe) discussions, [which] promote diversity and social justice learning by acknowledging the dynamics of oppression and privilege both inside and outside the classroom.”

Recognition of student diversity and the advantages of engaging with a diverse learning community was overwhelmingly present and therefore warranted a specialized focus within the larger discussion of Voice. Co-Construction of Knowledge was apparent through the repeated pedagogical strategy of “group” and “team” work, “class participation” and “discussions.” All of these ultimately aimed to design the class to be “interactive and participatory” and as one syllabus states, “enhance learning as students share their insights, perspectives, and experiences. Students will develop and refine their thoughts through the discussion process, plus broaden their classmates' understanding of the course content.” Some of the most overt forms of acknowledging the value of student input and agency was depicted through the policy sections. One syllabus read,

I will conduct this class in an atmosphere of mutual respect. I encourage your active participation in class discussions. Each of us may have strongly differing opinions on the various topics of class discussions. The conflict of ideas is encouraged and welcome, through civil discourse. The orderly questioning of the ideas of others, including mine, is similarly welcome.

Another read, “A best effort is made to provide an opportunity for students to comment on a proposed change before the change takes place.” These policies, though attending to different aspects of the course (i.e., curriculum and content debate vs. course expectations), both cultivate an intentionality toward student involvement and agency, capitalizing on diversity and differences as a mechanism for open discussion and learning.

### 3.4 Critical approaches

This theme denotes the deliberate intention to engage students in critical reflection to assess and understand their unique biases, worldviews, beliefs, etc., as well as “interrogate dominant cultural norms and assumptions as well as their own social positions” within current systems (13). Critical Approaches, similar to Voice, was originally developed as part of the PHCR praxis, but our analysis identified a unique facet of Critical Approaches warranting a new category. Advocacy was noted as a salient mechanism to engage students in the critical reflection described within Critical Approaches. Therefore, any prompt that had students engage in macro-level (i.e., political, social, and legal) support, recommendations, and/or activism for underserved and marginalized populations that is possible or strengthened by student's position in society was coded under Advocacy.

Critical Approaches appeared in a variety of ways, but one of the more unique was an instructor's request to, “Be open, listen, and ask questions. Avoid ‘blaming' statements. Respect opinions that you may not agree with. If someone says something that you feel is uncomfortable or offensive, take this as an opportunity to share your perspective.” In doing so, the instructor is calling for students to acknowledge and reflect on their unique perspectives, but also allow others to share theirs in an effort to collaborate and challenge one's assumptions. This was echoed in other syllabus language which discussed learning through group collaboration as,

Through collaboration, individual perspectives on ethically complex situations are developed, challenged, and refined. In public health work, the multitude of perspectives shape issues, options, and outcomes. Practicing team interactions around ethically sensitive issues, can provide you with a foundation for how to engage in these types of interactions in your career.

This sentiment presents students with an opportunity to not only reflect on their perspectives, but also recognize that redefining and evolving their perspectives is an organic part of public health work. In addition to transactional language regarding classroom conduct and culture, several syllabi utilized Critical Approaches within their assignment prompts. For example, one “self-reflection” assignment asked students to “Examine the ways in which students' own life experiences and perspectives inform their public health work and make a personal connection to the concept of equity.” This prompt requires students to engage in critical reflection to recognize how their unique lived experiences may be either perpetuating or dismantling systems of inequity.

Advocacy was an identified category within Critical Approaches that had students articulate support, recommendations, and/or activism on behalf of a vulnerable population. Among both PHPs and SPHs, one shared competency was prominent, “advocate for political, social or economic policies that will improve health in diverse populations.” This learning objective/course competency was met through various assessments including developing of an advocacy statement, authoring an op-ed, policy brief or public education material related to advocacy, and writing directly to a legislator. More specifically, for the advocacy statement, students were tasked with writing:

A brief description of the population…and its vulnerability and advocate for specific solutions to address the rights of the population in relation to its overall health status, disease prevention, access to healthcare services or treatment, and/or to addressing a specific determinant of health (e.g., education, social or racial discrimination, socioeconomic conditions, structural/organizational issues (e.g., lack of access to clean water, sanitation, transportation or other essential services, etc.)) that may influence health outcomes in this group.

### 3.5 Culture of inclusivity

This theme was one of two newly constructed *themes* based on inductive coding procedures. Culture of Inclusivity depicts an intentional effort to establish classroom and course expectations of respect, civility and openness to allow individuals to have open and authentic conversations and relationships among peers and instructors. Of note, this theme differs from the previous theme “Voice” because it does not aim to prioritize and/or center marginalized persons, but instead develop and foster a sense of community among and across racial groups to respect and uplift each other's experiences. It was salient across both PHPs and SPHs, but with a dominating presence within the policies sections of the syllabi. Within PHPs, approximately 94 percent of this theme was captured within the policies, and within SPHs, approximately 85 percent was within the policies. One syllabus overtly states that it intends to, “sustain an environment of inclusiveness that empowers us all to achieve our highest potential without fear of prejudice or bias.” Others offered similar sentiments such as,

All are welcome here. As a public health practitioner, I value diversity, equity, and inclusion. I look forward to working with each of you as you grow into future public health heroes. I will work hard to cultivate an inclusive environment where all of us can work toward success and support each other on our journey through this course. We will learn and grow together

or

I believe that learning happens best when we all learn together as a community. This means creating a space characterized by generous listening, adventurous civility, humility, patience, and hospitality. I will strive to create a safe classroom environment that promotes scholarly dialogue and informed debates that are respectful of diverse perspectives.

Some even pinpoint specific facets of inclusivity such as, “This course affirms people of all gender expressions and gender identities. If you prefer to be called a different name than what is indicated on the class roster, please let me know. Feel free to correct me on your preferred gender pronoun.” Interestingly though, a majority of academic syllabi outlined policies near the middle or end and were often the wordiest sections. Therefore, though this theme was salient, it may not be as impactful or effective among student populations actively engaging in the courses themselves since it can be easily missed amongst the other syllabus components.

### 3.6 Ordinariness of Racism

Ordinariness of Racism was operationalized as any explicit mention of racism's embeddedness within societal practices, routine engagements and everyday exposures regardless of its root (i.e., implicit bias, microaggressions, overt discrimination, etc.). There were just over two times as many accounts of this theme identified within PHPs compared to SPHs. This being said, the distribution across these two entities was fairly similar with Ordinariness of Racism predominantly being prevalent in course competencies and curricular information. One competency that was repeatedly noted, across both PHPs and SPHs, likely due to its relation to CEPH accreditation, was, “Discuss the means by which structural bias, social inequities and racism undermine health and create challenges to achieving health equity at organizational, community, and societal levels.” Within curricular information, there were a variety of assignments that addressed various facets of racism such as implicit bias or identifying the multi-level nature of racism. For example, one assignment requests that students, “identify a health outcome, associated risk factors at all levels of the SEM, and the morbidity and mortality of a specific population experiencing disparities that result from structural bias, social inequities, and racism.” Calling for students to focus on racism through the Social Ecological Model (44, 45) allows students to recognize the pervasiveness of racism at both micro and macro levels of society. Others asked students to actively engage with these concepts by reflecting on their own biases which may perpetuate the pervasive nature of racism such as, “Complete 1 Implicit Association Test.”

Finally, though there were not many instances within the other syllabus sections, some of the more thorough in addressing this theme were within the course overview section. Some syllabi set a strong situational precedent by acknowledging the Ordinariness of Racism within the current sociocultural and political climate. For example, one course overview included,

Given the events that have transpired since the onset of the COVID-19 pandemic and all the health inequities and injustices the pandemic has exacerbated and made even more visible and the social unrest as a result of violence and injustices perpetrated on US residents, particularly people of color; and given the long history of structural racism and its insidious effects, the course will utilize a social justice perspective to examine both historical and current issues with a focus on the underlying causes of these injustices and solutions to those problems.

Though this course overview speaks to specific events such as the onset of the COVID-19 pandemic, it is further calling upon its audience to acknowledge the deeply rooted and routine exposures that this pandemic onset simply exacerbated.

### 3.7 Social construction of knowledge

This theme was nearly equal between PHPs and SPHs with 37 and 34 accounts, respectively. It was defined as the recognition and determination that established knowledge is inherently subjective and tied to the social contexts in which it observed, analyzed, and presented. Interestingly though, Social Construction of Knowledge was not identified in either entity's policies. Rather, an overwhelming majority resided within the course competencies, objectives and goals. For example, learning objectives such as “Select quantitative and qualitative data collection methods appropriate for a given public health context,” and “Interpret results of data analysis for public health research, policy or practice,” both speak to the subjective nature of scientific knowledge due to context and receiving audience. Comparatively, “Synthesize evidence and anecdote from a variety of sources to inform and persuade,” demonstrates the subjectivity of information and knowledge based on one's intentionality behind developing and contextualizing literature to inform and persuade various groups.

Often when discussing the theme, Social Construction of Knowledge, within the curricular information sections of the syllabi, it was frequently posed as a reflection-based assignment allowing students to grapple with the concept of subjectivity. For example, one syllabus asks, “Why is it important to contextualize the health policy environment?” Another utilized a “Nested cultural humility assignment” where students were asked to select a health equity issue of their choice and reflect on any biases or assumptions they may have regarding this issue. They were then prompted to “engage in a short-term effort to challenge assumptions through independent learning. This independent learning can take many forms (book reading, community-based discussions, documentary viewing, following community advocates on social media, and many other potential methods).” The provided examples of independent learning allow the recipient to recognize the variety of valid information sources and knowledge bases that can facilitate student learning. Others echo a complimentary sentiment by establishing that student experiences and perspectives represent various valid forms of knowledge and should be used as a mechanism to work through complex situations: “Through collaboration, individual perspectives on ethically complex situations are developed, challenged, and refined. In public health work, the multitude of perspectives shape issues, options, and outcomes.”

### 3.8 Intersectionality

Intersectionality is defined as “the interlocking nature of multiple social identities (e.g., race, gender, sexual orientation, SES, disability) and the recognition of how they operate within current systems of privilege and oppression” (46–49). This theme was disproportionately distributed with a majority of accounts being present within PHPs, with nearly three times more occurrences compared to SPHs. Several examples of Intersectionality were identified within the course competencies, objectives and goals such as, “Reflect on your identities, intersectionality, cultural beliefs and values and how they manifest in your day-to-day life and relate to health” and “Analyze how social identities relate to inequities on both individual and societal levels.” These course objectives depict both explicit attention toward Intersectionality with the term being directly used within the objective, as well as an implicit operationalization of the construct by outlining the facets of intersectionality: (1) social identities, (2) oppression or privilege (i.e., inequities), and (3) their operation within larger society. Though there were several exemplary models within the course competencies, objectives, and goals, a majority of occurrences were found within the curricular information. More specifically, one course was dedicated to this concept, including it across the entire semester through assigned weekly readings such as, “Quantifying intersectionality: An important advancement for health inequity research” (50), “Evolving Intersectionality within Public Health: From Analysis to Action” (51), “Intersectionality in Public Health Research: A View From the National Institutes of Health” (52), and “Navigating the Storm: How to Apply Intersectionality to Public Health in Times of Crisis” (53). This degree of intentionality toward Intersectionality provides a wide breadth of exposure and engagement for students enrolled in this course.

### 3.9 Disciplinary self-critique

This theme had a total of 50 occurrences, with a nearly equal distribution across PHPs and SPHs. Disciplinary Self-Critique was defined as the systematic examination by members of a discipline to acknowledge and address racial inadvertences due to current discipline-specific efforts that perpetuate systems of inequality. Due to the operationalization to “examine,” 29 of the 50 accounts within this theme manifested in course competencies, objectives, and goals. Examples include, “Identify strengths and limitations of current public health models and approaches and design innovative solutions for public health challenges today and in the future,” “Describe how public policy both creates and solves public health problems,” and “…consider the business, demographic, ethnocultural, political, and regulatory implications of decisions and develop strategies that continually improve the long-term success and viability of the organization.” These learning objectives situate the conversation for students to grapple with all potential outcomes—both positive and negative, for a wide variety of populations. Interestingly, by the way these objectives are written, having students propose innovative solutions and/or strategies for “today and in the future” or “that continually improve the long-term success” implies that this should be an iterative process, requiring students to recurrently engage in critique and adjustment. Of note, though the course objectives section had a high prevalence of Disciplinary Self-Critique, this did not overwhelmingly translate into curricular information. That being said, when identified within the curricular information, several assignment descriptions and/or prompts strongly aligned with the operationalization of this theme, such as, “Consider the role of the public health system in reinforcing the health inequity including the following: (policies, programs, resource flows, power dynamics, relationships, and mental models) reinforce the current health inequity” or “Identify unintended consequences of public health system changes that could occur as the result of your proposed approach.”

### 3.10 Primacy of racialization

Primacy of Racialization was defined as the fundamental contribution of race as an organizing construct to establish ordered, hierarchical systems within society. This theme was identified over three times as many times within PHPs compared to SPHs. Unlike other themes, identification of Primacy or Racialization has a negative connotation because it demonstrates public health curriculum's perpetuation of reifying racial boundaries and attributing negative health outcomes to *race* rather than drawing attention to the systematic origin of health inequities due to *racism*. The identified occurrences are not expansive, yet they are overt. For example, one reading was described as “Review: Data Summaries by Population. Black/African Americans” and was continued throughout the semester to also include “American Indians,” “Asians” and “Hispanics/Latinos.” Other accounts were identified within the course competencies, objectives and goals such as “Understand the role of race, ethnicity, gender, and other individual characteristics in descriptive epidemiology.” Again, this objective continues to draw discrete racial boundaries, as well as boundaries around other social identities, as a means of communicating and inferring epidemiological data.

### 3.11 Race as a social construct

This theme was the second least prevalent theme. Race as a Social Construct was operationalized as an acknowledgment or mention of a subjectively produced “racial significance that derives from social, political, and historical forces” that is ultimately perpetuated by societal practices, systems, and underlying norms (13). One of the more overt demonstrations of this theme was stated within the policy sections of various syllabus such as,

Additionally, how you identify in terms of your gender, race, class, sexuality, religion, and dis/ability, among all aspects of your identity, is your choice whether to disclose (e.g., should it come up in classroom conversation about our experiences and perspectives) and should be self-identified, not presumed or imposed,

or “Unwelcome conduct directed toward another person based upon that person's actual or perceived race, actual or perceived gender, color, religion, age, national origin, ethnicity, disability, or veteran status, or for any other reason, may constitute a violation of…” Utilizing language such as “not presumed or imposed” and “actual or perceived” outlines the subjective nature of race, and other social identities, and how these are socially constructed based on the collective perception of our peers and ourselves. Complementary to this policy, one PHP syllabus assignment prompt asks students to detail the “social construction of target populations” within their case study. This specific and intentional use of language is offered likely in lieu of what we often refer to as demographic information. By contextualizing this as social construction, it directly speaks to the operationalization of this theme.

### 3.12 Race consciousness

Race Consciousness was the least prevalent theme with only three accounts noted throughout the data. This theme was defined as a “deep awareness of one's racial positions” (13), as well as an awareness of racial dynamics in social, interpersonal, and intrapersonal contexts. One of these three accounts was a “nested cultural humility assignment,” which prefaced student work with the following statement,

There exist unspoken social “scripts” we hold for “The Other” in relation to ourselves that reflect these identities. These scripts impact our relationships and our effectiveness in fulfilling our mission. If we can recognize those scripts, we have the opportunity to interrupt them, and potentially transform them before we negatively impact someone, or reify and reproduce inequality.

The assignment then indicated that there would be a class activity and students would afterward reflect on the activity to assess which identity scripts they were most aware of, and how identifying these scrips helped in their personal development, relationships and efforts to advance health equity. In contrast to an assignment, one account resided within the course overview where the following statement was included, “George Floyd, Breonna Tayler, Ahmaud Aubrey, Rayshard Brooks, Dontre Hamilton, Tony Robinson, Renisha McBride, and too many Black human beings before and after them all had their right to live taken away from them before they were wrongfully and unjustly killed.” In doing so, this instructor drew attention to the racial identities of the outlined individuals, likely catalyzing individual racial reflection. Furthermore, by including a recent event, this statement drew upon timely sociocultural and sociopolitical conversations to provide relatable and relevant contexts for their student audience.

## 4 Discussion

A significant portion of public health efforts involve serving historically marginalized communities to increase accessibility to health services and reduce negative health outcomes. However, public health has underutilized CRT to serve ethnic and racial minority communities. However, in recent years, there has been an opportunity for public health educators to incorporate race criticality within public health education to train the next cohort of public health professionals in this realm of thought, philosophy and practice. This study aimed to be the first to investigate public health education's current curricular practices in relation to CRT and anti-racist praxes through an evaluative document analysis of CEPH-accredited MPH programs' syllabi.

Our results demonstrate that all principles of the PHCR praxis were present, along with four other salient constructs. Interestingly though was the variability in prevalence across themes. For example, the most prevalent theme was Structural Determinism. A seemingly complementary theme, Intersectionality, had a fraction of the number of cases as Structural Determinism within the analyzed syllabi. The two themes are representing two facets of the same conversation due to the original construct of intersectionality being conceived out of one of the most pervasive structural determinants—the legal system (49). This finding is particularly curious because it may depict a failure to connect and contextualize the two innately interwoven concepts and depict how people are uniquely a product of and influence on the social determinants via our intersectional identities. Others have openly noted that discussing systems of oppression and intersection of identity markers cannot be fully examined independently (54). Therefore, it is essential to provide a holistic lens when discussing the pervasiveness of structural determinism, which innately includes intersectionality. By continuing to present these constructs as neatly bound from one another, we are failing to depict the true nature and pervasiveness of racism and its effect on marginalized and minoritized populations.

In contrast to the most prevalent theme, Race Consciousness was the least prevalent with only three occurrences total. This was surprising due to the visual depiction of the PHCR praxis having Race Consciousness as the overlaying lens that all operations should occur through (13). As such, one would expect to see it significantly more throughout our curriculum. The construct of Race Consciousness was brought to the forefront of critical race studies to directly combat the era of colorblindness and to dismantle the perceived “taboo” nature of race and racial awareness (55, 56). Furthermore, a push for race consciousness is essential in dismantling the synonymous nature of “Whiteness” and “normalcy” within American society (9). However, this dismantling approach is reliant upon White individuals rejecting their White identity, relinquishing their privilege (57), and demonstrating openness to reformation (58). Specifically, regarding reformation, it is assumed that by bringing awareness of Whiteness to White individuals, they will be more inclusive toward and accepting of other cultures (59). Ultimately, this requires White individuals to think about, reflect upon, and dismantle their White privilege (57), thus pointing to the necessity of race consciousness across all individuals.

Within public health, race consciousness has started to emerge as a top-of-mind construct within health disparities work. More specifically, several examples of conducting race conscious efforts have been noted within endometrial cancer (60), palliative care (61), cardiovascular health (62) and pediatric care research (63). This may be due to, as Markovich (62) states, the “double whammy of racial awareness and reckoning in the United States” in 2020 which not only warrants but requires an expansive and intentional race consciousness within public health (p. 1). However, in alignment with our results, race consciousness within public health educational efforts has not been widely published. In fact, there is an overwhelming lack of literature addressing race consciousness within any disciplinary learning environment.

Within our analysis, there were also several new themes and categories developed. Interestingly, the two new categories were developed under a pre-existing PHCR praxis theme, offering a specialized focus that was uniquely prominent across the dataset. One category, Co-construction of Knowledge, was especially prominent, so much so that quantitatively exceeded its superordinate theme, Voice. This category focused on both implicit and explicit recognitions of the value of student diversification due to the unique perspectives and individual personal and professional experiences as a facet of constructing course discussion and knowledge. This is particularly interesting since a recent 2023 amendment to the CEPH criteria states that, “the school or program makes efforts to include diverse voices and perspectives from a range of students in these decision-making structures” (64), which are expectantly reported through descriptive self-studies that specifically satisfy this requirement. Noting the benefits of student diversity throughout course activities may be one way to self-report fulfilling this call. Some have quite literally taken to the idea of co-constructing knowledge by working with students to develop course exams (65), while others have expressed that empowerment and agency cannot be adequately addressed through individual attribute or assignments alone. Instead, a comprehensive evaluation and mindfulness toward the sociocultural contexts in which we aim to cultivate student-centered learning and agency is also required (66). This dualistic approach was present with our data with the simultaneous presence of another inductively developed theme—Culture of Inclusivity.

Often, Co-Construction of Knowledge was presented as student group projects and classroom discussion, which allows students an opportunity to hear from and work with their peers. Through this group participation and collaboration, students can engage in social perspective taking, or the opportunity to view a situation or experience from another person's perspective (67). Researchers have stipulated that it is vital that in today's multiculturally diverse classroom makeups, it is more important now than ever before to prioritize and foster social perspective taking to capitalize on each other's experiences as learning opportunities (68, 69). However, it is essential to also include components of Culture of Inclusivity to achieve reciprocal and student-based learning rather than being a source of conflict or tension or reduction of students of color personal experiences and stories being used and commodified to benefit White students' learning (70). Potentially more important though is that nurturing social perspective taking via co-constructing knowledge, as well as fostering the skills necessary to operate within an inclusive culture and community directly applies to public health students since our students will not only engage with diverse groups within their programs but are expected to serve diverse populations in the community. As such, predominantly White public health programs may require additional considerations on how to translate this intended class exercise into co-constructing knowledge with the communities they intend to serve. Thus, the higher prevalence of both Co-Construction of Knowledge and Culture of Inclusivity compared to other themes leaves an optimistic impression in the way we are training and educating students to interact with diverse groups and populations of people.

### 4.1 Limitations

There are several limitations to consider when interpreting the results of this study. First, non-response bias. Though effort was made to minimize sampling bias, several institutions were unresponsive and therefore there may be a potential difference in the institutions that responded and were willing to share their academic syllabi vs. the institutions that did not respond or declined to participate. Secondly, the paradigmatic nature of qualitative inquiry may inadvertently lead to bias, particularly within the data analysis procedures. However, the utilization of an existing methodological framework within the directed content analysis procedures and the use of multiple, independent coders from varying sociodemographic backgrounds likely reduce the influence of bias on the results. Finally, it is recognized that some of these principles may be present within a course, even though it is not stated within the course syllabus. Future studies should evaluate the entirety of a course, ranging from document analysis of the syllabus and e-learning system materials to classroom observations to get a more holistic snapshot of what is occurring within MPH courses.

It is recognized that this research serves as a first step to a series of work that is required to make substantial change in public health education, regarding inclusiveness and recognition of the value and importance of CRT. As such, this study strictly sought to assess whether instructors are providing MPH students with opportunities to engage with racial tenets and antiracist tenets, not whether students are successfully achieving the intended competencies. Future research is needed to evaluate whether students are demonstrating these PHCRP principles in a successful and meaningful manner. Additionally, further investigations into our identified race consciousness deficiency are warranted. Being the overlaying principle, of which the rest of reside within, is superficially concerning. It is hypothesized that this is likely due to a majority of public health faculty and instructors identifying as White or Caucasian, with slow progress toward diversification (71). This then requires us to ask, how could public health education adequately explore alternative epistemologies when it has been historically developed and grounded in Eurocentric traditions? Thus, we should consider that Whiteness itself could be the limiting factor in progress toward transforming public health education to be antiracist, further perpetuating systematic White ignorance (72). This is likely further exacerbated by the fact that faculty have potentially failed to consider their White privilege, recognize White supremacy, and name White dominance. As Mills (72) states, “the white delusion of racial superiority insulates itself against refutation,” thus perpetuating the socialization of epistemology to be grounded in Eurocentric norms. Without disrupting the cognitive tendency or state of white ignorance, no such reform of social construction of knowledge or critical approach to examine its appropriateness is possible. This inherently may be the source of the limited number of race consciousness exemplars identified within this research study.

Regarding frequencies, investigation into the distribution across specific institutions was not conducted for this study. Exploring whether individual institutions are more holistically addressing these principles compared to others, though all have met the accreditation threshold, may be worthwhile. It may even call into question how we currently accredit and if a more multifaceted and multi-modal approach is warranted to truly address these competencies. Finally, the political landscape within recent years has demonstrated hostility toward CRT, diversity, equity, inclusion, antiracism, and social justice at academic institutions. Our study did not account for state political affiliation or related legislation. Future research comparing syllabi pre- anti-DEI legislation compared to post may be advantageous to identify any strategic language shifts, political affiliation state trends, and differences between public and private institutions.

### 4.2 Conclusion

In conclusion, to the best of our knowledge, this study is the first to investigate public health education's current curricular practices in relation to CRT and antiracist praxes. It is essential that we recognize that public health education prefaces our students' future practices as public health professionals. More specifically, public health education situates and prioritizes the topics and competencies we consider to be foundational knowledge and skills for our students to have upon program completion. As such, we need to continue to evaluate whether our educational efforts align with the overarching public health disciplinary goals and objectives. This study provides a means to evaluate the current use of CRT tenets and antiracist principles to meet CEPH's “diversity” criterion for accreditation. Our findings suggest that several themes are being well attended to and widely acknowledged across programs; however, our results also evince that there are still several gaps in which we, as educators, can continue to work on and foster throughout our courses. Ultimately, the identified presence and prevalence of each theme provides useful information for Master of Public Health programs to adapt, adjust, or reinforce their current curricular, instructional and pedagogical efforts to align with the overarching discipline's goal to achieve health and racial equity.

## Data Availability

The data analyzed in this study is subject to the following licenses/restrictions: the dataset will not be made available to maintain confidentiality and anonymity of participating institutions. Requests to access these datasets should be directed to Sarah L. Collins, sarahcollins@ku.edu.
